# Highly Wear-Resistant Triboelectric Nanogenerators Based on Fluorocarbon-Graphene Hybrids

**DOI:** 10.3390/nano15100763

**Published:** 2025-05-19

**Authors:** Ke Zhang, Liang Zhang, Jinlong Ren, Yubin Li, Zaibang Wu, Kaihan Shan, Lin Zhang, Lingyu Wan, Tao Lin

**Affiliations:** 1 School of Physical Science and Technology, Guangxi University, Nanning 530004, China; 2207301175@st.gxu.edu.cn (K.Z.); 2207301173@st.gxu.edu.cn (L.Z.); renjinlong@st.gxu.edu.cn (J.R.); 2307401020@st.gxu.edu.cn (Y.L.); 2207301150@st.gxu.edu.cn (Z.W.); 2207301017@st.gxu.edu.cn (K.S.); 2207301176@st.gxu.edu.cn (L.Z.); 2 Laboratory of Optoelectronic Materials and Detection Technology, Guangxi Key Laboratory for Relativistic Astrophysics, Guangxi University, Nanning 530004, China; 3 Center on Nanoenergy Research, Guangxi Colleges and Universities Key Laboratory of Blue Energy and Systems Integration, Guangxi University, Nanning 530004, China; 4 Guangxi Key Laboratory of Electrochemical Energy Materials, Guangxi Novel Battery Materials Research Center of Engineering Technology, State Key Laboratory of Featured Metal Materials and Life-Cycle Safety for Composite Structures, Guangxi University, Nanning 530004, China

**Keywords:** triboelectric nanogenerators, fluorinated ethylene vinyl ether, three-dimensional hierarchical porous graphene, wear resistance

## Abstract

Triboelectric nanogenerators (TENGs) are pivotal for powering small electronic devices by converting mechanical energy into electrical energy. However, the wear resistance of TENG friction layers remains a critical barrier to their long-term performance. This study introduces a hybrid material combining fluorinated ethylene vinyl ether (FEVE) and three-dimensional hierarchical porous graphene (3D HPG) to address these challenges. FEVE was selected for its low friction coefficient and excellent wear resistance, while 3D HPG enhances charge generation and transfer efficiency. The incorporation of 3D HPG into FEVE significantly improves both triboelectric output and durability, achieving a charge density of 140 μC/m^2^, surpassing conventional copper-based TENGs (50–120 μC/m^2^). The hybrid material demonstrates minimal performance degradation over 10^5^ sliding cycles, highlighting its potential for durable, low-cost, and high-efficiency TENGs in wearable and portable electronics.

## 1. Introduction

Sufficient energy is a fundamental requirement for human survival and development. Efficient energy harvesting and transmission have long been crucial topics due to the ever-growing demand for energy. In particular, with advancements in information technology and artificial intelligence, a vast number of electronic and optoelectronic devices are now integrated into human environments. These functional devices are expected to connect and exchange information, enabling intelligent decision-making and replacing humans in various tasks, ultimately forming the so-called “Internet of Things” (IoT). With the development of 5G and more advanced wireless signal transmission technologies, high-speed connectivity between these devices has largely been achieved. However, efficiently powering these devices remains a significant challenge, as they are often miniaturized and, in some cases, even placed on moving carriers, such as the human body. Traditional rechargeable batteries are increasingly unable to meet the demands of miniaturization and long-term sustainability. To address this issue, the concept of “self-powering” has been proposed [1,2,3]. This approach involves harvesting energy from irregular environmental motions-such as mechanical vibrations, body movements, or water waves-to generate electricity and directly power electronic devices. The realization of self-powering is based on the triboelectric nanogenerator (TENG) [4], which operates by bringing two materials into contact. Due to the irregular motion in the environment, these materials either slide against each other or repeatedly come into contact and separate. During these interactions, electrons transfer between the materials due to their differing triboelectric properties, which determine their tendency to gain or lose electrons. As a result, one material becomes positively charged while the other becomes negatively charged. The accumulated charges generate an electric field, inducing electron flow through an external circuit and producing electrical current.

Over the years, researchers have made significant advancements in enhancing the efficiency of TENGs through various strategies. These include optimizing material design to maximize surface charge density for transfer [5,6], refining device structures to improve charge transfer efficiency [7,8], and enhancing circuit designs to boost energy harvesting efficiency [9,10]. However, TENGs inevitably suffer from friction layer abrasion, leading to continuous performance degradation and limiting their practical lifespan. A critical challenge remains how to significantly improve the wear resistance of TENG friction materials without compromising output power density. To address this issue, there is growing interest in developing superior materials for friction layers. However, previous studies have revealed a persistent trade-off between triboelectric performance and durability [11,12]. An alternative approach is to enhance the triboelectric performance of highly wear-resistant materials. In particular, the introduction of micro/nano composites with superior electrical properties has proven to be an effective strategy [13,14,15,16,17,18,19,20,21,22,23,24,25,26,27,28,29,30,31,32,33,34,35,36,37,38,39,40]. Graphene, renowned for its exceptional electrical and mechanical properties, has emerged as a promising material for hybrid TENGs [19,20,21,41,42,43,44,45]. Early research by Wang et al. [17] and Liu et al. [18] demonstrated graphene’s charge storage capabilities, significantly expanding its potential applications in TENGs. Since 2014, Kim et al. [21] have developed the first transparent, flexible graphene-based TENG, inspiring numerous subsequent advancements. These graphene-based TENGs have demonstrated improvements in short-circuit current, open-circuit voltage, and surface charge density. However, the original objective of these studies was primarily to enhance the electrical performance of established triboelectric layers (e.g., PTFE) through graphene doping or coating [21,23,30,46,47,48,49,50,51]. Due to the intrinsically soft macroscale properties of graphene, devices employing graphene-enhanced electrical outputs frequently exhibit compromised operational stability under prolonged use. A representative example is the work by Ping Yang et al. [52], wherein graphene nanoplatelets were integrated into PTFE to improve TENG output stability. Their experimental results revealed a 17.7% decline in short-circuit current and a 16.6% reduction in open-circuit voltage following 1.5 × 10^5^ contact-separation cycles. These investigations, while advancing electrical output metrics, have yet to sufficiently resolve the durability limitations inherent to graphene-enhanced TENG systems.

In this study, we propose a novel approach to enhance the durability of TENGs by incorporating fluorinated ethylene vinyl ether (FEVE), a type of fluorocarbon resin, as the positive triboelectric layer (PTL). FEVE exhibits low friction and excellent wear resistance [53,54,55]. However, when used alone as a replacement for conventional copper foil, its improper electronegativity leads to a decline in charge generation performance. To overcome this limitation, we fabricated and integrated a three-dimensional hierarchical porous graphene-like (3D HPG) material as a functional filler within the FEVE layer. The study demonstrates that, unlike other polymer host materials, FEVE exhibits exceptional suitability as a host for graphene, attributed to the steric hindrance generated by fluorocarbon polymers between graphene sheets, which effectively suppresses their agglomeration tendencies. Furthermore, the incorporation of 3D HPG into the system significantly enhances charge generation and transfer processes in the TENG, ultimately leading to improved electrical output performance. Importantly, the unique cage-like, porous structure plays a crucial role in optimizing triboelectric performance, as the large number of edge-exposed carbon atoms on the surface possess a remarkable capacity for capturing and storing net charges. The resulting output charge density in air reaches 140 μC/m^2^, surpassing the reported values (50–120 μC/m^2^) [56] achieved by conventional PTFE-metal TENGs and approaching the theoretical limitation of air breakdown. Detailed information comparing this study with existing studies in the field is given in Table S1 of the Supporting Materials. More importantly, the addition of 3D HPG reduces friction and enhances the wear resistance of the FEVE layer. This improvement is attributed to the lubricating properties of the layered 3D HPG structure and the rapid recovery of local deformation in the FEVE matrix. These findings highlight the potential of 3D HPG-FEVE hybrid material as an effective PTL for developing low-cost, high-efficiency TENGs with extended operational lifespans.

## 2. Experimental

### 2.1. Material

The 3D HPG was synthesized following a previously reported method [57]. The 3D HPG material exhibits an ultrahigh specific surface area (1810 m^2^ g^−1^) that generates substantial capacitance through abundant ion adsorption sites. Its submicrometer-scale macropores function as ion reservoirs while minimizing diffusion distances, collectively enhancing charge transfer kinetics and reducing interfacial resistance. The integrated micro-meso-macroporous network facilitates rapid ion transport, enabling exceptional rate capability. Combined with graphene-like conductivity and electrochemical stability, this architecture synergistically achieves high-rate operation and ultralong cycling durability. FEVE was sourced from Shanghai Guihong Industrial Co., Ltd. (Shanghai, China), while the multi-layered graphene (MG) aqueous conductive slurry was obtained from Suzhou Tanfeng Graphene Technology Co., Ltd. (Suzhou, China) Additional materials, including fluorocarbon diluents, curing agents, driers, and surfactants, were acquired from commercial suppliers in the Chinese domestic market. Copper foil, polytetrafluoroethylene (PTFE) films, acrylic sheets, sponge sheets, and conductive wires were also procured from local suppliers for device assembly.

### 2.2. Preparation of 3D HPG-FEVE Complex

Initially, 50 mL of FEVE coating was poured into a beaker, and varying masses of 3D HPG were weighed and dispersed into the solution with an appropriate volume of fluorocarbon diluent to prepare 3D HPG-FEVE mixtures with mass fractions of 0.1, 0.5, 1, 1.5, and 2 wt%. The mixtures were magnetically stirred at 60 °C for 4 h, followed by ultrasonic homogenization for 30 min. Next, the solutions were transferred into a planetary ball-milling container and processed at 330 rpm for 1 h. After ball milling, a controlled amount of fluorocarbon curing agent was added to the mixtures. The final solutions were then magnetically stirred at 60 °C for 5 min, subjected to an additional 5-min ultrasonic treatment, and vacuum-dried at 70 °C for 1 h.

### 2.3. Preparation of Contact-Separation TENG and Sliding TENG

Acrylic sheets were laser-cut to the designated dimensions (3 cm × 6 cm) with pre-drilled holes for wire connections, ensuring a flat surface. The negative triboelectric layer (NTL) was assembled by sequentially laminating a sponge, copper foil, and a PTFE film. Meanwhile, the prepared composite solution was uniformly coated onto another prefabricated copper foil/sponge/PTFE plate using a film applicator, serving as the PTL. The coated layer of PTL was air-dried at an ambient temperature for 7 days, frozen at −20 °C for 12 h, and subsequently freeze-dried under vacuum for 6 h.

Electrical wires were connected to the electrode layers of both triboelectric components, enabling the final integration of PTFE/3D HPG-FEVE-based TENG devices in both contact-separation mode and sliding-mode configurations. Details of the process flow are given in Supplementary Note S1 of the Supporting Materials.

### 2.4. Characterization Methods

The X-ray diffraction (XRD) data were collected using an X-ray diffraction system (Bruker D8 Discover, Billerica, MA, USA) with a Cu Kα radiation source. Raman scattering spectra were acquired using a Raman spectrometer (Horiba iHR550, Kyoto, Japan). Surface roughness of the samples was measured using atomic force microscopy (AFM, Bruker Dimension Icon, Malaysia). Fourier transform infrared (FTIR) spectroscopy data were obtained using an FTIR spectrometer (IRTracer-100, Kyoto Japan). The morphology and microstructure of the 3D HPG and 3D HPG-FEVE samples were characterized using scanning electron microscopy (SEM, JSM-6510A; Zeiss Sigma500, Jena, Thuringia, Germany), and elemental distribution was analyzed using an attached energy-dispersive X-ray spectroscopy (EDS) system. Fatigue testing was performed using an electro-hydraulic servo fatigue testing machine (EMT-5KNV-50, Kyoto, Japan). The samples were secured using clamps equipped with force and displacement sensors, and subjected to high-frequency, long-duration friction testing. Data analysis and export were conducted using the accompanying Servo4830 4.08 operating software.

## 3. Result and Discussion

### 3.1. Fabrication of 3D HPG-FEVE Triboelectric Layers

**Figure 1:**
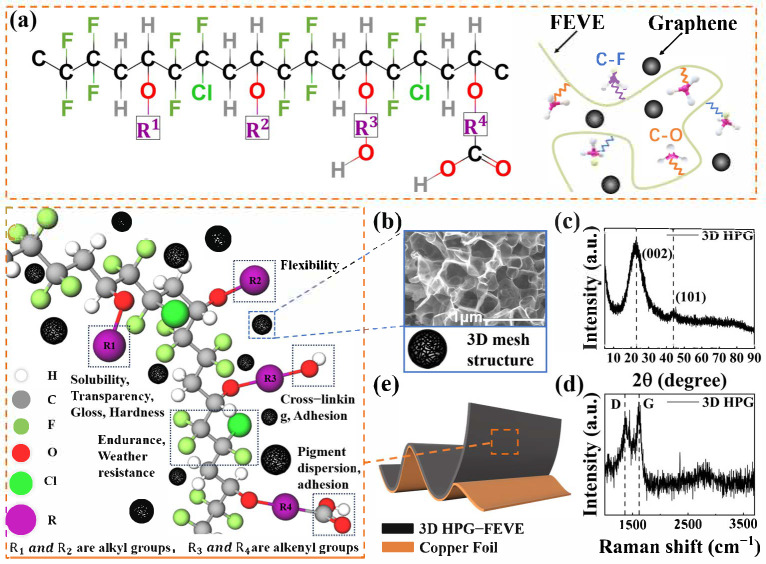
Schematic diagram of the structure of 3D HPG-FEVE nanocomposite coating: (**a**) FEVE molecular formula, 3D HPG-FEVE molecular chain diagram and molecular structure diagram, (**b**) SEM image, (**c**) XRD image, and (**d**) Raman scattering spectrum of 3D HPG, (**e**) Schematic diagram of 3D HPG-FEVE PTL.

Figure 1a illustrates the basic structure of the FEVE polymer used in this study. This copolymer consists of alternating sequences of fluorinated olefins and specific vinyl ether units, forming a completely amorphous structure. The alternating sequence provides exceptional weather resistance, while the combination of various vinyl ether comonomers imparts key properties such as solubility in organic solvents, crosslinking reactivity, strong adhesion, film hardness, and flexibility. FEVE is composed of numerous C–F bonds with high bond energy. Due to the high electronegativity of fluorine atoms, repulsion occurs between adjacent fluorine atoms, causing the fluorine atoms in fluorocarbon chains to be helically distributed along the zigzag carbon backbone. The carbon chain is surrounded by negatively charged fluorine atoms, and due to their symmetrical distribution, the entire molecule remains nonpolar. The low polarizability of fluorine gives FEVE excellent insulating properties, exceptional thermal stability, and chemical inertness. Notably, the molecular structure, featuring strong C–F bonds and a fluorine-shielded carbon backbone, creates a slick, waxy surface that reduces shear forces during movement, ensuring consistent lubricity across a wide range of temperatures and chemical environments. In this study, 3D HPG was incorporated into the FEVE framework as a filler to enhance its electrical properties. As shown in the SEM image (Figure 1b) and XRD patterns (Figure 1c), the applied 3D HPG contains abundant micro- and mesopores composed of graphene walls. The Raman scattering spectrum in Figure 1d further supports these structural characteristics, displaying two distinct peaks at 1350 cm^−1^ (D peak) and 1610 cm^−1^ (G peak). In graphene, the G peak corresponds to the lattice vibrations of carbon atoms after sp2 hybridization, while the D peak indicates lattice defects. The intensity ratio of I (D)/I (G) in the spectrum reflects an sp2 cluster size in the micrometer range. This graphene-like structure provides excellent conductivity and high electrochemical stability, while the 3D porous network offers a large surface area, resulting in enhanced capacitance. Ideally, due to the high spatial hindrance effect of the folded FEVE long chains, 3D HPG can be uniformly dispersed within the voids surrounding these chains, forming a compact composite. The integration of 3D HPG is expected to improve the conductivity of the complex. Consequently, the mixture is coated onto the surface of copper foil to form an enhanced PTL, as illustrated in Figure 1e.

**Figure 2:**
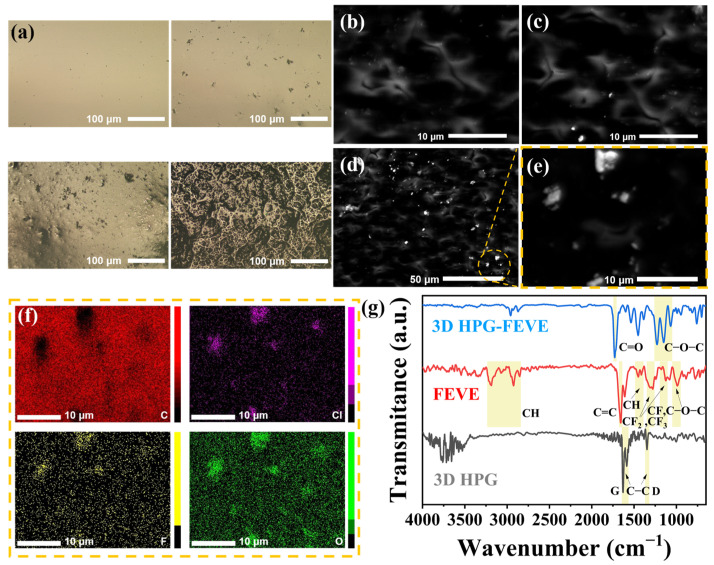
3D HPG-FEVE morphology and structure characterization: (**a**) Optical surface morphology of 3D HPG-FEVE coating samples with different mass fractions (0, 1, 1.5 and 2 wt%), (**b**–**d**) SEM images of 3D HPG-FEVE composite coatings with different mass fractions (0.1, 0.5 and 1 wt%), (**e**) EDS measurement area, (**f**) EDS image of 3D HPG-FEVE composite coating, (**g**) FTIR spectrum of 3D HPG-FEVE composite coating.

The surface morphology of FEVE samples with different 3D HPG doping concentrations was observed using an optical microscope. As shown in Figure 2a, the pure FEVE coating exhibits a clean and smooth surface. This characteristic is preserved after doping with 1 wt% 3D HPG, with only a small number of inhomogeneous precipitates distributed on the surface. However, increasing the doping concentration beyond 1 wt% results in a noticeable increase in surface roughness due to the agglomeration of 3D HPG. The XRD analysis in Figure S1a supports this observation, as several sharp characteristic peaks associated with the graphite phase appear in samples with 3D HPG concentrations exceeding 1 wt%. The FTIR analyses (Figure S1b) further reveal that the signal intensities related to the partially oxidized graphite phase increase with higher 3D HPG concentrations. At a doping level of 2 wt%, the sample exhibits a fully porous surface, indicating the breakdown of the FEVE framework. These results suggest that the critical doping concentration of 3D HPG is approximately 1 wt%. The surface morphology of samples with 0.1, 0.5, and 1 wt% was further characterized by SEM. Figure 2b–d reveals bright spot precipitates on otherwise smooth surfaces. Atomic force microscopy (AFM) measurements (Figure S2, Supporting Information) indicate an average roughness of ~10 nm for smooth regions. A smaller precipitate-rich area was analyzed via EDS, as shown in Figure 2e,f. Elemental mapping confirms uniform carbon distribution in smooth areas, verifying effective 3D HPG incorporation into the FEVE matrix. In contrast, precipitates exhibit reduced carbon content but elevated fluorine, chlorine, and oxygen levels characteristic of FEVE polymers. These results confirm the precipitates as FEVE aggregates rather than 3D HPG. FTIR spectra are provided in Figure 2g. The pure 3D HPG exhibits a strong characteristic peak at 1630 cm^−1^ with a small shoulder at 1580 cm^−1^, corresponding to the in-plane stretching vibration of sp2-hybridized carbon atoms in the graphene lattice, commonly referred to as the G band. Another prominent peak at 1350 cm^−1^ represents the bending vibration of C–C bonds, known as the D band. For pure FEVE, the strong peak at 1660 cm^−1^ corresponds to the stretching vibration of fluorinated C=C bonds, attributed to unpolymerized fluorinated vinyl groups. A series of characteristic peaks in the 1100–1400 cm^−1^ range corresponds to C–F bond stretching vibrations, including asymmetric stretching (1250–1350 cm^−1^) and symmetric stretching (1100–1200 cm^−1^) of CF_2_ or CF_3_ groups. The 950–1050 cm^−1^ band is associated with C–F bending vibrations or skeletal vibrations of ether bonds (C–O–C). Additionally, the 2800–3200 cm^−1^ and 1400–1500 cm^−1^ bands correspond to the stretching and bending vibrations of C–H groups, respectively. The FTIR spectrum of FEVE mixed with 1 wt% 3D HPG exhibits significant differences compared to pure FEVE. The presence of both G and D bands confirms the incorporation of 3D HPG. However, the peak at 1660 cm^−1^ is noticeably suppressed, suggesting the weakening of fluorinated C=C bond stretching. Instead, new strong peaks appear at 1720 cm^−1^, 1250 cm^−1^, 1150 cm^−1^, and 1050 cm^−1^, which are commonly observed in oxidized graphene (GO) materials. Specifically, the peak at 1720 cm^−1^ corresponds to C=O stretching, while the 1050–1250 cm^−1^ peaks are associated with epoxy group (C–O–C) stretching. These results indicate partial oxidation of 3D HPG during the mixing process. Additionally, the incorporated 3D HPG may react with residual fluorinated vinyl in the system, leading to the disappearance of the C=C bond stretching signal. Overall, these results confirm the uniform distribution of 3D HPG clusters within the FEVE matrix. The polymer network remains intact as long as the doping concentration does not exceed 1.5 wt%.

### 3.2. Triboelectric Performance of 3D HPG-FEVE Layers

There are two typical configurations of TENGs designed for different motion scenarios: contact-separation (CS) mode and sliding mode. Their basic architecture and operational principles are illustrated in Figure S3 in Supporting Information. To ensure broader applicability, it is essential to evaluate the triboelectric performance of the obtained 3D HPG-FEVE as the PTL in both configurations. In both architectures, a PTFE layer serves as the NTL, given its strong tendency to acquire electrons during contact-separation or sliding. Meanwhile, 3D HPG-FEVE functions as the PTL due to its strong tendency to lose electrons. Both the thin PTFE layer and the 3D HPG-FEVE layer are coated onto copper foil and supported by a rigid acrylic plate. The copper foils act as electrodes, connecting to external circuits for electricity collection. When the triboelectric layers come into contact, triboelectrification induces positive charges on the 3D HPG-FEVE surface and an equal number of negative charges on the PTFE surface. When an applied force causes longitudinal separation (CS mode) or lateral separation (sliding mode), the charge equilibrium between the triboelectric layers is disrupted, creating a potential difference. This results in electron flow from the copper foil on the PTFE side to the copper foil on the 3D HPG-FEVE side through the external circuit, generating a pulse of positive current. When the layers move back into contact, the charge equilibrium is disrupted again, reversing the electron flow and producing a pulse of negative current.

**Figure 3:**
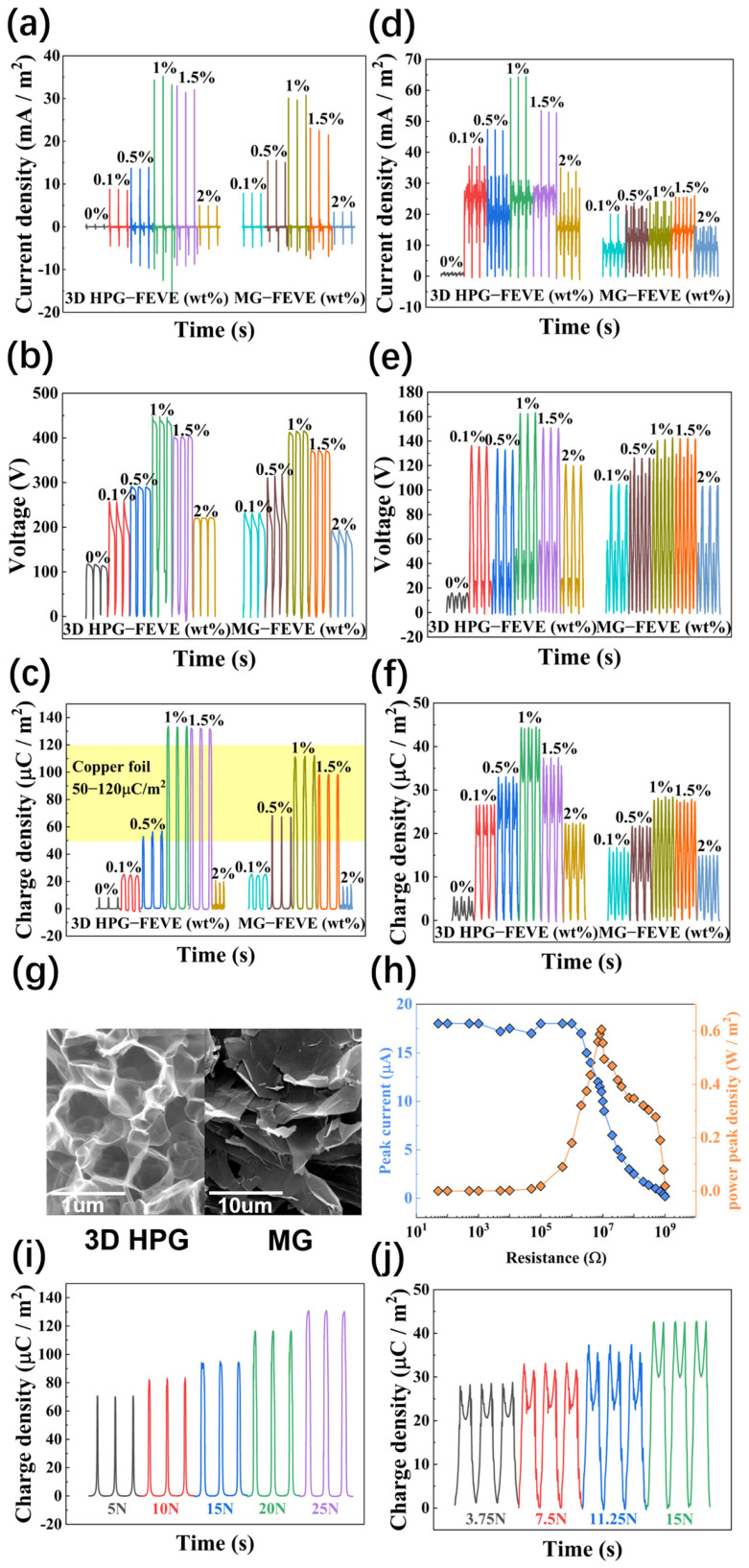
Characterization of electrical output performance of 3D HPG-FEVE TENGs: (**a**–**c**) Current, voltage, and charge of TENG with different PTLs in contact-separation mode, (**d**–**f**) Current, voltage, and charge of TENG with different PTLs in sliding friction mode, (**g**) Comparison of SEM images of 3D HPG and MG, (**h**) 1% 3D HPG-FEVE TENG electrical output characteristics diagram, (**i**) Output charge densities of 1% 3D HPG contact-separation TENG under different forces, (**j**) Output charge densities of 1% 3D HPG sliding TENG under different forces.

Figure 3a–f present the output performance of PTFE/3D HPG-FEVE-based TENGs in CS mode and sliding mode, respectively. According to the triboelectric principle, the output voltage, output current density, and harvested charge in each cycle are all proportional to the electrostatic-induced charges on the triboelectric layer surfaces. As a result, these parameters evolve consistently with increasing 3D HPG doping concentration in FEVE, indicating enhanced charge transfer efficiency in the 3D HPG-FEVE/PTFE contact. Theoretically, charge transfer efficiency depends on the electronegativity difference between the two triboelectric layers. Fluoropolymer-based materials like PTFE and polyvinylidene difluoride (PVDF) are commonly used as NTLs due to their extremely high electronegativity. Conversely, insulating polymers with lower electronegativity, such as silk, nylon, and cotton fiber, are used as PTLs. Alternatively, highly conductive metals such as copper, aluminum, or steel are employed as PTLs due to their high carrier mobility and low work function, which contribute to their relatively low electronegativity in triboelectric material series. The combination of copper foil and PTFE is one of the most widely used TENG architectures due to its low cost and ease of fabrication. Previous studies have reported output charge densities for copper-PTFE-based TENGs ranging from 50 to 120 μC/m^2^ under atmospheric conditions [56], serving as a benchmark for PTFE/3D HPG-FEVE-based TENGs (indicated by the yellow zone in Figure 3c). In our study, the combination of pure FEVE and PTFE exhibits a relatively low output charge density (<5 μC/m^2^) due to FEVE’s high electronegativity, which results in a small electronegativity difference with the PTFE layer. However, incorporating 3D HPG significantly enhances output performance, increasing the charge density to 140 μC/m^2^ in CS mode at a 1 wt% doping concentration-exceeding the reported values of copper-PTFE-based TENGs. Similarly, the sliding-mode TENGs show a maximum charge density of 70 μC/m^2^ at 1 wt% doping. These results suggest that 3D HPG lowers the electronegativity of the FEVE polymer, likely due to its semimetallic nature and conductivity comparable to copper. Additionally, the unique porous structure of 3D HPG plays a crucial role in charge transfer. To examine this, we compared the triboelectric performance of another graphene-like material, multi-layered graphene (MG), as a filler in FEVE. MG shares a similar component and crystalline structure related to graphene with 3D HPG. However, it mainly consists of 6–10 graphene layers with an average lateral size of 50 μm, which is outside the 3D mesh structure (Figures S4 and S5). As shown in Figure 3a–f, despite having higher conductivity than 3D HPG, MG-FEVE exhibits noticeably worse triboelectric performance under identical fabrication conditions. This discrepancy is attributed to MG’s lack of a 3D porous structure. Prior to device fabrication, we prepared control FEVE films without graphene additives. The presence of F⁻ ions in FEVE leads to its relatively high electronegativity, yet the resultant TENG devices exhibited poor electrical outputs with transferred charge density below 5 μC/m^2^. Given graphene’s inherent semiconductor properties and higher intrinsic electronegativity relative to FEVE, our experimental results confirm that optimized 3D HPG incorporation significantly enhances device performance. We acknowledge that not only the ability of losing/gathering electrons, which depends on the electron affinity and work function of the triboelectric layers, but also the ability of storing charges on their surface plays significant roles in determining the output performance of TENGs. Previous electrostatic studies have shown that carbon atoms near the edges and corners of graphene sheets have a significantly higher capacity to capture or store net charges than those in the central region [17]. The 3D HPG structure, with its extensive graphene sheet bending and cage-like morphology, exposes a greater number of edges on the contacting surface compared to flask-stacked graphene layers. These edge-exposed carbon atoms make a major contribution to surface charge density. FEVE PTLs with doping concentrations exceeding 1 wt% exhibit lower output charge densities for both 3D HPG- and MG-doped samples. Based on the characterization results, this decline is due to graphene agglomeration and the breakdown of the FEVE framework, leading to a softer and rougher surface. This surface morphology is disadvantageous for electron harvesting because the agglomerated graphene clusters are prone to detachment, contaminating the opposing layer. Meanwhile, numerous studies have previously reported enhancing TENG performance through graphene-based electrodes or doping strategies, but excessive addition of graphene materials can lead to aggregation phenomena, ultimately limiting further improvement in electrical output. Through systematic research and experiments, we found that FEVE materials can effectively reduce such aggregation, enabling uniform dispersion of graphene within the film. This allows the incorporation of a significant amount of graphene, thereby imparting the device with excellent electrical output performance. Furthermore, as demonstrated in Figure 3h, the electrical output performance of the 1 wt% 3D HPG-FEVE TENG device was evaluated under varying load conditions, revealing a maximum power density of 6.05 W/m^2^ when connected to a 9 MΩ external resistor. The influence of applied force on triboelectric output was systematically investigated. Figure 3i,j illustrate that in contact-separation mode, increasing the applied force from 5 N to 25 N elevated the output charge density from 70 μC/m^2^ to 140 μC/m^2^. Correspondingly, in sliding mode, augmenting the applied force from 3.75 N to 15 N enhanced the charge density from 28 μC/m^2^ to 43 μC/m^2^. This phenomenon can be attributed to improved contact intimacy under higher pressure, particularly between surfaces with nanometer-to-micrometer-scale roughness [58]. These results highlight the importance of wear resistance in triboelectric layers, as higher applied pressure enhances output power but also accelerates material abrasion.

### 3.3. Wear Resistance of 3D HPG-FEVE Triboelectric Layer

**Figure 4:**
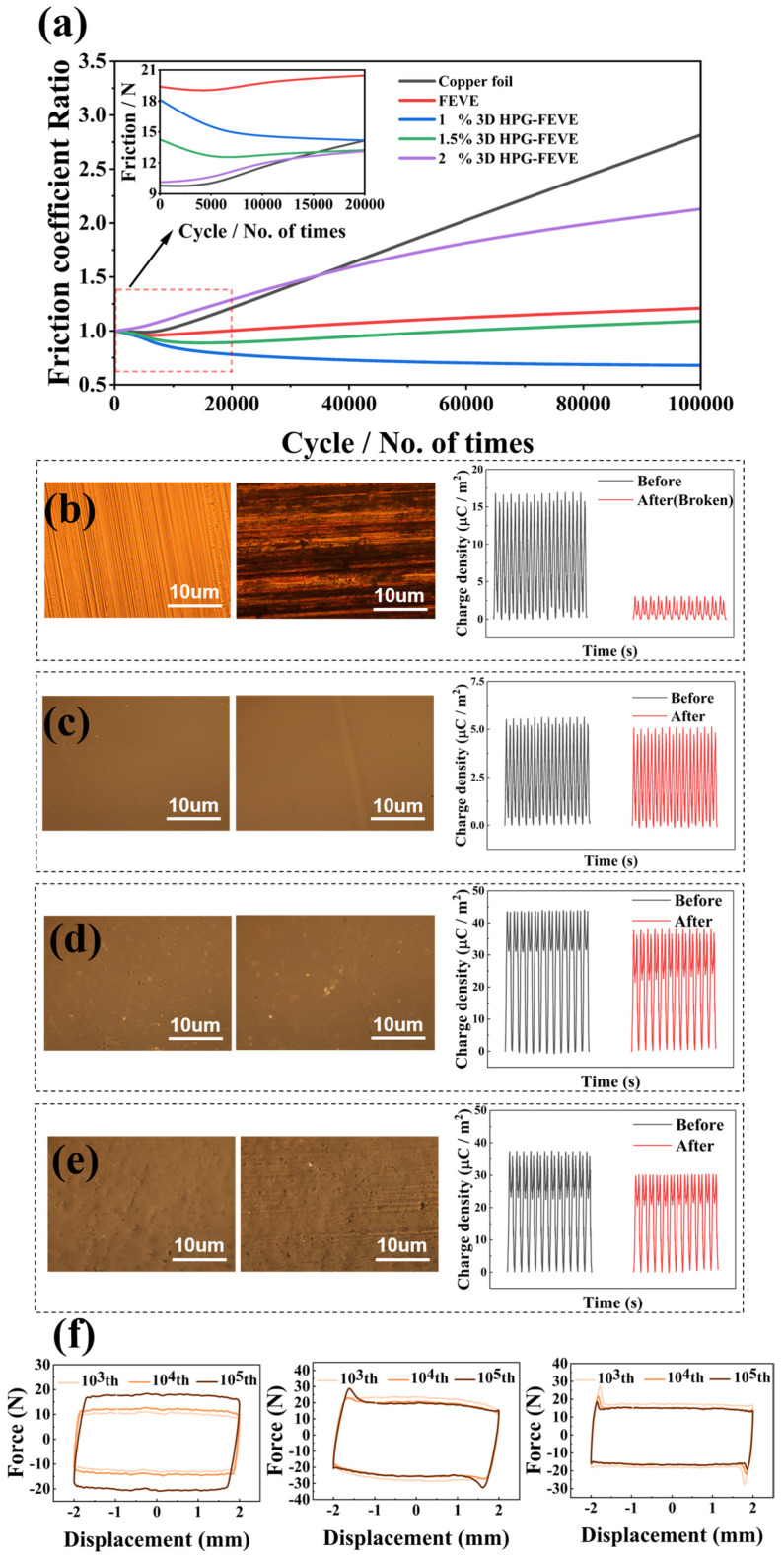
Characterization of wear resistance of 3D HPG-FEVE PTLs: (**a**) Changes of the friction coefficient compared to the initial values of different PTLs under 10^5^-cycle fatigue tests. The inset presents the frictional force in the first 20,000 cycles. Optical micrographs of the (**b**) copper foil surface, (**c**) FEVE surface, (**d**) 1 wt% 3D HPG-FEVE surface, and (**e**) 1.5 wt% 3D HPG-FEVE surface before and after fatigue testing, and the change in output charge density of the TENGs. (**f**) Friction force-displacement characteristic curves of copper foil, FEVE, and 1 wt% 3D HPG-FEVE at the 10^3^th, 10^4^th, and 10^5^th cycle during fatigue tests.

In practical applications, ensuring the long-term durability of a TENG is far more critical and challenging than achieving short-term ultra-high output performance, especially in scenarios requiring sliding-mode operation. Over time, the contacting surfaces of the two triboelectric layers inevitably undergo abrasion, leading to increased surface roughness, macroscopic scratches, and cross-contamination. These factors contribute to a gradual decline in output performance. Therefore, two synergistic aspects, the stability of the electrical output performance and the stability of surface roughness, were applied to quantitatively evaluate the device durability. The surface friction coefficient tests are used to support the examination of surface roughness. Fatigue tests were conducted on FEVE layers with varying concentrations of 3D HPG, serving as PTLs in sliding-mode TENGs. During each test, a 3D HPG-FEVE layer was subjected to reciprocating sliding motion against a PTFE layer under constant applied pressure. The friction coefficient was calculated as the ratio of the measured frictional force to the applied vertical force. As shown in Figure 4a, the obtained friction coefficients were normalized to their respective initial values at cycle zero. The inset of Figure 4a compares the initial frictional properties of different samples. Copper foil exhibits a significantly lower initial friction coefficient than pure FEVE film due to its hard and polished surface. However, incorporating increasing concentrations of 3D HPG into FEVE progressively reduces the friction coefficient. This effect is attributed to the graphite-like, layered structure of 3D HPG, which enhances surface lubrication. Notably, effective lubrication alone does not ensure long-term stability of low friction, as wear resistance also depends on the structural integrity of the FEVE matrix. The results show that the friction coefficient of copper foil increases dramatically-by a factor of 3.5-after 10^5^ sliding cycles. Optical micrographs in Figure 4b reveal the formation of deep, groove-shaped scratches on the surface. This structural degradation correlates with a significant drop in output charge density, from 15 μC/m^2^ to less than 2 μC/m^2^, indicating that deep scratches severely impair charge collection. In contrast, the pure FEVE layer maintains a nearly constant friction coefficient over 10^5^ sliding cycles, with no observable changes in surface morphology under optical microscopy. Correspondingly, its triboelectric performance remains stable, with only a 5% decline in output charge density. This high resistance to frictional damage is attributed to the compact carbon backbone of FEVE and its stable C–F bonds. However, the overall output charge density remains low, consistently ranging between 5.2 and 5.5 μC/m^2^, due to FEVE’s improper electronegativity. The addition of 3D HPG significantly improves performance. FEVE layers doped by 1 wt% 3D HPG exhibit not only lower initial friction coefficients than pure FEVE but also a counterintuitive reduction in friction coefficients over extended sliding cycles. Optical micrographs confirm that no significant scratches form on these surfaces, and output performance declines by only 10%, demonstrating excellent long-term stability. Taking previous surface morphology results (Figure 2d) into consideration, the initial roughness of 3D HPG-FEVE layers is attributed to the presence of precipitates formed during mixing. These precipitates are gradually polished away during sliding, resulting in a smoother surface over time—a phenomenon validated through detailed surface roughness measurements via AFM technique (Figure S6). Concurrently, the surface resilience-deformation relationship was characterized by AFM (Figure S7). With DMT modulus values greater than 100 GPa for copper foil versus less than 20 GPa for FEVE-based materials, the enhanced deformation recovery capacity of the polymer composites relative to metallic substrates confirms minimal impact of the polishing process on electrical output performance. However, excessive 3D HPG doping leads to structural degradation of the FEVE matrix, making the surface softer and less compact. This leads to the irreversible material migration and shedding over time, which weakens the wear resistance. As shown in the micrograph, FEVE layers with 1.5 wt% 3D HPG develop ordered scratches after prolonged sliding, accompanied by a more pronounced decline in output charge density. This phenomenon is even more severe in samples with 2 wt% doping, further validating the hypothesis that excessive 3D HPG content compromises structural integrity and long-term performance.

To further investigate the intrinsic causes of the improved lubricating properties and wear resistance in 3D HPG-FEVE layers, the variation of frictional force within a single sliding cycle was analyzed. As shown in Figure 4f, the frictional force-displacement loop of copper foil maintains a regular parallelogram shape, indicating that the frictional force remains nearly constant within each cycle. However, as the number of cycles increases to 10^5^, the magnitude of friction gradually rises. Given that increased surface roughness is typically associated with the formation of macroscopic scratches, this feature suggests that surface scratches appear instantly and permanently as sliding occurs. In contrast, the pure FEVE layer exhibits an irregular frictional force loop characterized by a distinct peak at maximum displacement (both positive and negative), where the sliding direction reverses. Following each peak, the frictional force decreases temporarily before stabilizing until the next directional change. This behavior arises from the transition between static and sliding friction, during which the FEVE surface undergoes deformation and recovery. Unlike the instantaneous and permanent scratch formation observed in rigid copper foil, this deformation-recovery mechanism contributes to FEVE’s high wear resistance, which is attributed to its compact yet flexible molecular structure. More importantly, in the FEVE-1 wt% 3D HPG composite, the frictional force peaks are sharper compared to those in pure FEVE. This observation suggests that the recovery process occurs more rapidly, as the sliding motion between graphene layers replaces the need for FEVE matrix deformation, thereby dominating the friction process. This result confirms the role of 3D HPG in enhancing surface lubrication. Overall, these findings demonstrate that the incorporation of 3D HPG not only improves the triboelectric performance of FEVE by enhancing charge transfer efficiency but also significantly enhances its wear resistance by lubricating the surface and mitigating the formation of detrimental surface damage.

## 4. Conclusions

In summary, this study introduces a novel approach to enhancing both the durability and efficiency of Triboelectric Nanogenerators (TENGs) by integrating FEVE and 3D HPG as the PTL. The hybrid material combines the wear resistance of FEVE with the charge-enhancing properties of 3D HPG, achieving a remarkable charge density of 140 μC/m^2^ in contact-separation (CS) mode and 70 μC/m^2^ in sliding mode. The output performance of the device, evaluated by the collected charge density over one cycle, demonstrated outstanding longevity, with only a 5% degradation after 10^5^ sliding cycles. In-depth analysis revealed that the excellent wear resistance of FEVE is attributed to its rapid recovery from local deformations rather than the formation of permanent scratches. The addition of 3D HPG further enhances this feature, thanks to its superior lubricating properties, resulting in improved overall durability. These results address the critical trade-off between triboelectric performance and durability, positioning 3D HPG-FEVE hybrids as a transformative material for self-powered IoT devices, wearable electronics, and industrial sensors.

## Data Availability

The raw data supporting the conclusions of this article will be made available by the authors on request.

## Supplementary Materials

The following supporting information can be downloaded at: https://www.mdpi.com/article/10.3390/nano15100763/s1, Table S1: Summary and comparison of the triboelectric materials for TENGs.; Figure S1: (a) XRD pattern of 3D HPG-FEVE, (b) FTIR images of different samples, (a) XRD pattern of 3D HPG.; Figure S2: AFM images: (a) AFM images of 3DHPG (inset: Graphene thickness), (b) average roughness of smooth surface areas.; Figure S3: Working principle of TENG: (a) contact-separation mode, (b) sliding friction mode.; Figure S4: SEM images with different magnifications (A–C) and TEM images (D–F) of the 3D HPG networks. (G) Low- and (H) high-resolution TEM images of the mesoporous texture of the few-layer graphene wall. The presence of small size mesopores ranging from 2 to 5 nm are clearly observed (some are indicated by the white arrows).; Figure S5: Comparison of structural characterization of MG graphene materials. (a) SEM image of MG, (b) TEM image of MG, (c) AFM image of MG, (d) XRD image of MG.; Figure S6: Average surface roughness and cycle number curves of different samples.; Figure S7: Rebound force-type variable characteristic curves of different sample surfaces.; Supplementary Note S1: Device process flow and the influence of different process parameters. References [31,57,59,60,61,62,63,64,65,66,67,68] are cited in the Supplementary Materials.
