# Supplementary material for: Highly Wear-Resistant Triboelectric Nanogenerators Based on Fluorocarbon-Graphene Hybrids
Source: Nanomaterials (Basel). 2025 May 19;15(10):763. doi: 10.3390/nano15100763 (PMC12113873; doi:10.3390/nano15100763)
Supplement: Supplementary file 1 [file nanomaterials-15-00763-s001.zip › nanomaterials-3581682-supplementary.pdf]

Supporting Information

Highly Wear-Resistant Triboelectric Nanogenerators Based on Fluorocarbon-Graphene Hybrids

Ke Zhang[1,2,3], Liang Zhang[1,4], Jinlong Ren[1,3,4], Yubin Li[1,4], Zaibang Wu[1,3], Kaihan Shan[1,2], Lin Zhang[1,2,3], Lingyu Wan [1,2,3], Tao Lin [1,2,3*]*

[1]School of Physical Science and Technology, Guangxi University, Nanning 530004, China

[2]Laboratory of Optoelectronic Materials and Detection Technology, Guangxi Key Laboratory for Relativistic Astrophysics, Guangxi University, Nanning 530004, China

[3]Center on Nanoenergy Research, Guangxi Colleges and Universities Key Laboratory of Blue Energy and Systems Integration, Guangxi University, Nanning 530004, China

[4]Guangxi Key Laboratory of Electrochemical Energy Materials, Guangxi Novel Battery Materials Research Center of Engineering Technology, State Key Laboratory of Featured Metal Materials and Life-cycle Safety for Composite Structures, Guangxi University, Nanning 530004, China

**Correspondence: correspondence to E-mail: lyw2017@gxu.edu.cn; taolin@gxu.edu.cn;*

Supplementary Figures

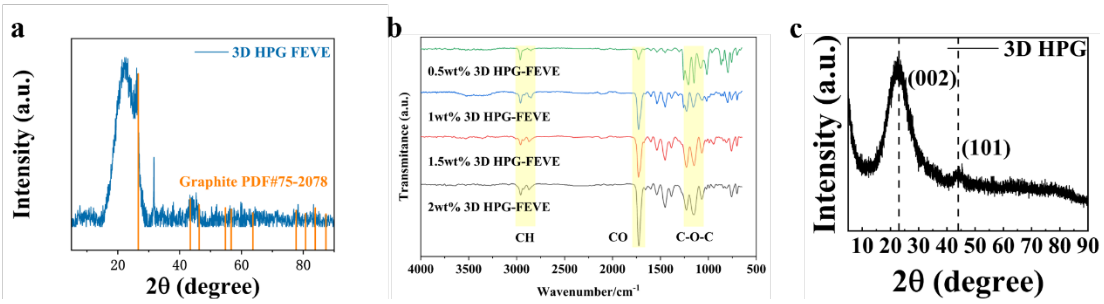

Figure S1 (a) XRD pattern of 3D HPG-FEVE, (b) FTIR images of different samples, (a) XRD pattern of 3D HPG

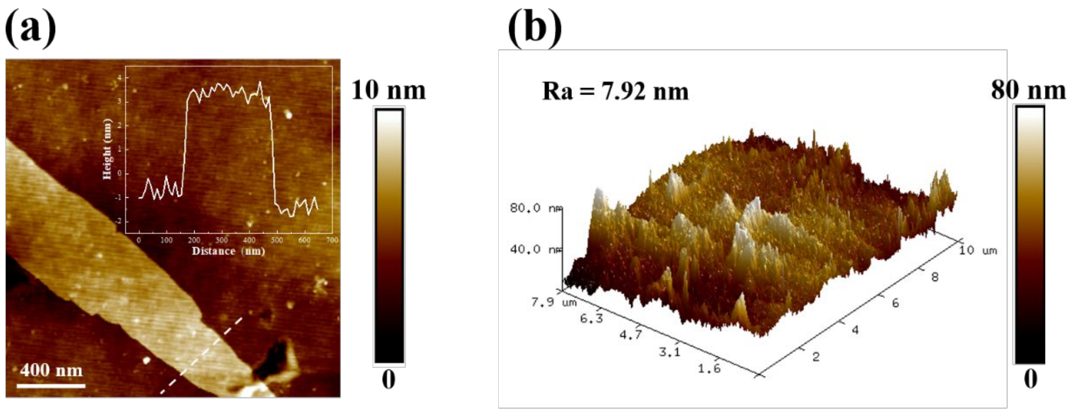

Figure S2 AFM images: (a) AFM images of 3DHPG (inset: Graphene thickness), (b) average roughness of smooth surface areas.

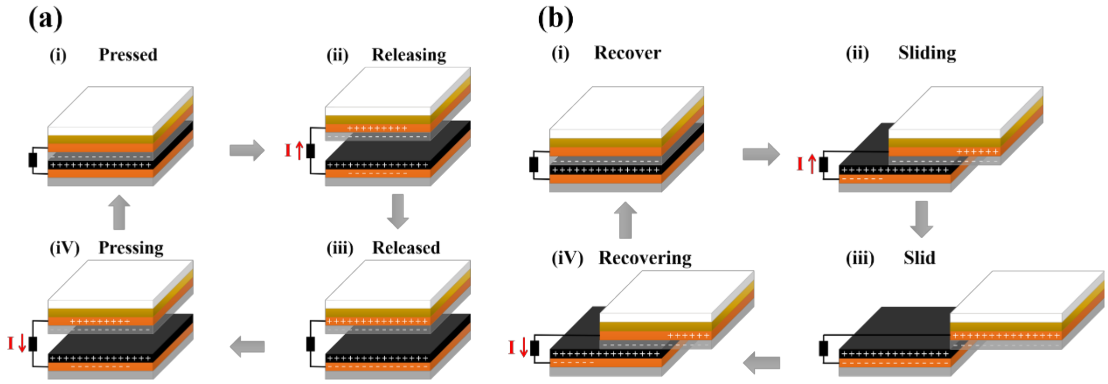

Figure S3 Working principle of TENG: (a) contact-separation mode, (b) sliding friction mode.

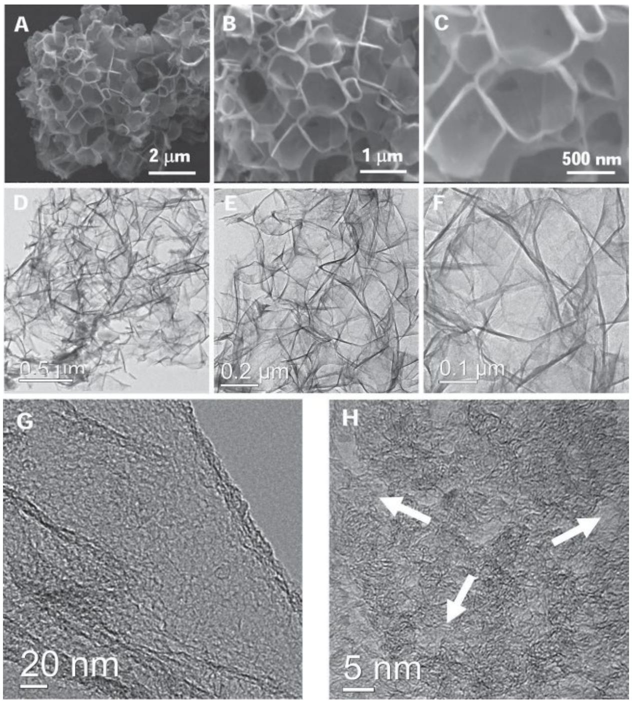

Figure S4[1] SEM images with different magnifications (A–C) and TEM images (D–F) of the 3D HPG networks. (G) Low- and (H) high-resolution TEM images of the mesoporous texture of the few-layer graphene wall. The presence of small size mesopores ranging from 2 to 5 nm are clearly observed (some are indicated by the white arrows).

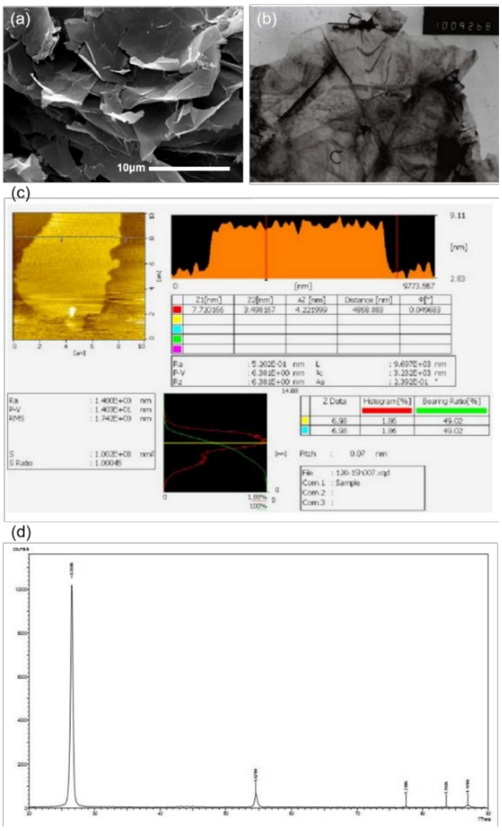

Figure S5 Comparison of structural characterization of MG graphene materials. (a) SEM image of MG, (b) TEM image of MG, (c) AFM image of MG, (d) XRD image of MG

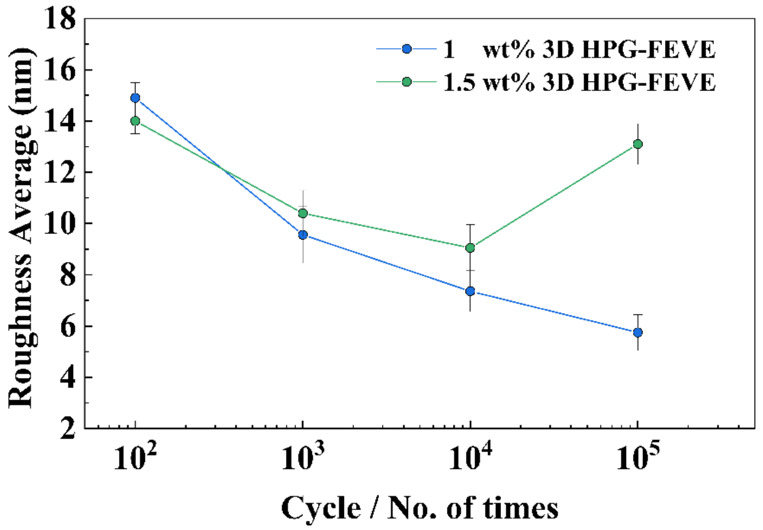

Figure S6 Average surface roughness and cycle number curves of different samples

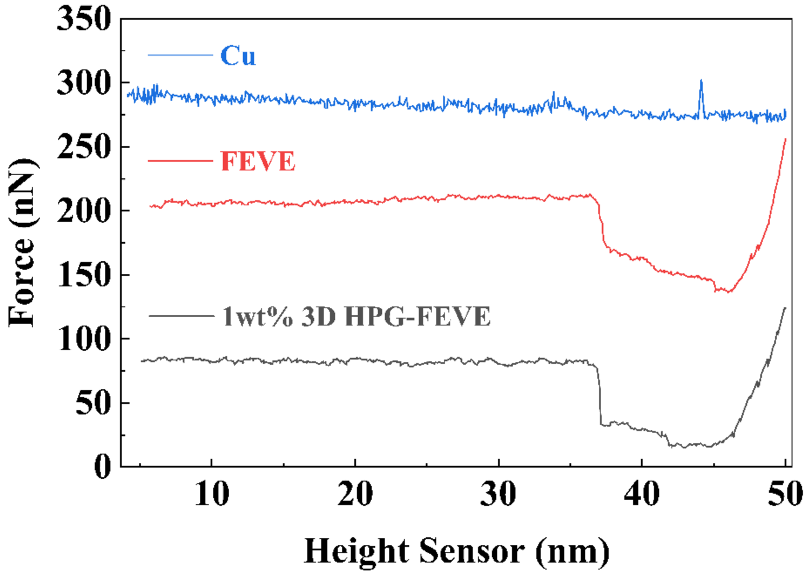

Figure S7 Rebound force-type variable characteristic curves of different sample surfaces

Table S1 Summary and comparison of the triboelectric materials for TENGs

Operating mode	Negative triboelectric materials	Positive triboelectric materials	Device dimension (cm)	Voltage (V)	Current density (mAm^{-2})	Charge density (μCm^{-2})	References
**contact-separation**	PTFE	3D HPG FEVE	3.0×6.0	450	35	140	This work
**sliding**	PTFE	3D HPG FEVE	3.0×6.0	160	65	43	This work
**contact-separation**	PTFE	Cu	5.0×5.0	20	60	120	[2]
**contact-separation**	PDMS/PTFE	Al	2.4×1.0	150	1.50	1	[3]
**contact-separation**	PTFE	polymer, polypyrrole(PPy)	1.7×1.7	28	28	84	[4]
**contact-separation**	Fluorinated ethylene propylene(FEP)	self-assembled monolayers	4.5×4.5	240	1.75	51	[5]
**contact-separation**	SiO_2	Cu	4.5×4.5	—	—	82.3	[6]
**contact-separation**	PVDF-ZnSnO3	polyamide-6 (PA6, Nylon)	9.0×9.0	520	2.7	62	[7]
**contact-separation**	PVDF-BTO	PA6	2.0×2.0/ 5.0×5.0	900	10	34.4	[8]
**contact-separation**	MSN-intercalatedMXene /PDMS	Al	—	461	12.1	—	[9]
**contact-separation**	MXene/PTFE	Cu	5.0×5.0	397	8.4	92.8	[10]
**contact-separation**	MXeneSrTiO3	poly-L-lysine(PLL)modifiedNylon11	2.0×2.0	137	8.5	—	[11]
**contact-separation**	PTFE	CNFs/MXene	$8cm^2$	24.9	2.01	15	[12]

Supplementary Note S1.

Device process flow and the influence of different process parameters

Each step and specific parameter in preparing 3D HPG-FEVE blended samples was optimized through extensive repeated experiments. First, pre-weighed FEVE coating material was placed in a beaker, followed by adding an appropriate amount of pre-synthesized 3D HPG powder. A critical step involved introducing a fluorocarbon diluent (primarily composed of butyl ester) into the mixture, followed by magnetic stirring and ultrasonication. Graphene powder exhibits hydrophobicity and aggregation tendencies. The butyl ester, as a solvent with moderate polarity, penetrates graphene aggregates, reduces surface tension, and enhances wettability with fluorocarbon resin. The inherent viscosity of fluorocarbon resin is mitigated by butyl ester addition, lowering system viscosity to facilitate graphene dispersion under shear forces during stirring, thereby preventing inhomogeneous dispersion caused by excessive viscosity. The reduced viscosity also improves energy transfer during ultrasonication, enhancing the fragmentation efficiency of graphene agglomerates. During heated stirring, the homogeneous solution of butyl ester and resin acts as a dispersion medium, achieving preliminary graphene uniformity and reducing subsequent ball-milling challenges. Heating simultaneously softens the fluorocarbon resin, enhances butyl ester solubility to promote resin-graphene interfacial bonding, and accelerates butyl ester diffusion into graphene interlayers, further exfoliating agglomerates and providing a more uniform initial dispersion state for ball milling. Additionally, butyl ester forms a solvation layer on graphene surfaces, reducing van der Waals forces between particles to inhibit post-ultrasonication/stirring re-agglomeration. Its compatibility with fluorocarbon resin ensures stable graphene dispersion within the resin matrix, preventing phase separation. The mixture was magnetically stirred at 60°C for 4 h to ensure homogeneity, followed by 30-min ultrasonication to eliminate bubbles and residual agglomerates.

The blended sample was then equally distributed into two ball-milling jars and processed in a planetary ball mill. The jars' combined planetary rotation and self-rotation generate high-energy collisions, shear forces, and friction. The complex three-dimensional trajectories of grinding balls thoroughly disrupt graphene agglomerates, achieving uniform dispersion in the fluorocarbon resin. Mechanical forces during milling create localized defects or active sites on graphene surfaces, strengthening chemical bonding/physical adsorption with fluorocarbon polymer chains, thereby improving mechanical properties and interfacial stability. Residual butyl ester acts as a lubricant, reducing friction between grinding balls and jars while maintaining system fluidity. Uniformly dispersed graphene forms a continuous network, significantly enhancing coating conductivity and thermal conductivity. The tight graphene-resin integration improves coating hardness, wear resistance, and impact resistance. Graphene's porous structure and chemical inertness further inhibit

corrosive medium penetration, synergizing with fluorocarbon resin to enhance corrosion resistance. Through iterative testing, ball milling at 330 rpm for 1 h was identified as optimal to prevent 3D HPG porous structure collapse or layer fracture.

Isocyanate groups in the fluorocarbon curing agent react with hydroxyl or other active groups in the resin to initiate crosslinking. Magnetic stirring ensures uniform curing agent dispersion via mild shear forces, preventing localized concentration spikes that could cause uneven curing or runaway reactions. Heating reduces slurry viscosity, facilitating bubble buoyancy and rupture, while mechanical stirring expels larger bubbles. Brief additional ultrasonication minimizes coating defects (e.g., pinholes, shrinkage pores), improves surface smoothness and corrosion resistance, and achieves submicron-level homogeneity by compensating for magnetic stirring limitations. The slurry was then vacuum-dried to lower the boiling point of residual butyl ester, accelerating its volatilization to prevent bubble formation or weak interfaces during curing. Vacuum conditions efficiently remove moisture and low-boiling impurities, promote tighter molecular chain packing, reduce internal porosity, and form dense prepolymers. Gentle heating induces preliminary resin crosslinking, increasing slurry viscosity and reducing fluidity for subsequent coating processes.

For TENG device fabrication, acrylic plates were laser-cut to specified dimensions (3 × 6 cm). The negative triboelectric layer (NTL) was assembled by sequentially laminating sponge, copper foil, and PTFE film, with sponge added to increase contact area. The positive triboelectric layer (PTL) was prepared by blade-coating the 3D HPG-FEVE composite solution onto a prefabricated copper-clad acrylic plate using a doctor blade with a 200 μm gap. The blade was positioned on PTFE/acrylic guide rails matching the substrate thickness to enable direct coating. Electrical connections to copper electrodes on both triboelectric layers completed the integration of PTFE/3D HPG-FEVE-based TENG devices in contact-separation and sliding-mode configurations.

PTL fabrication must commence immediately after slurry preparation to prevent excessive viscosity from compromising coating quality. The blade-coating process extended 2–3 mm beyond copper foil edges to counteract edge thinning caused by surface tension. Coated PTLs were air-dried at room temperature for 7 days under controlled humidity (excessive humidity causes condensation; low humidity induces electrostatic impurity adsorption). Samples were frozen at −20°C for 12 h, followed by 6 h of vacuum freeze-drying. Freezing immobilizes resin polymer chains, preventing graphene sedimentation/agglomeration due to density differences. Differential thermal contraction between copper foil and fluorocarbon resin releases interfacial stress during freezing, mitigating post-curing crack risks. Trapped bubbles shrink and stabilize under freezing, preventing defect expansion during drying. Immediate transfer to the freeze dryer after freezing minimizes surface melting. Sublimation of ice crystals during freeze-drying bypasses liquid phases, preserving graphene's 3D porous structure by avoiding thermal damage from high-temperature drying. Solvent removal under vacuum allows gradual resin chain relaxation, forming densely packed structures. Direct graphene-resin interfacial contact enhances van der Waals forces and chemical bonding by eliminating solvent barriers. Compared to

thermal drying, freeze-drying's slow sublimation enables molecular chain rearrangement, preventing internal stress concentration from rapid shrinkage.

1. Gao, Y.; Liu, G.; Bu, T.; Liu, Y.; Qi, Y.; Xie, Y.; Xu, S.; Deng, W.; Yang, W.; Zhang, C. MXene based mechanically and electrically enhanced film for triboelectric nanogenerator. *Nano Res.* **2021**, *14*, 4833–4840. https://doi.org/10.1007/s12274-021-3437-5.
2. Li, Y.; Li, Z.; Shen, P.K. Simultaneous Formation of Ultrahigh Surface Area and Three-Dimensional Hierarchical Porous Graphene-Like Networks for Fast and Highly Stable Supercapacitors. *Adv. Mater.* **2013**, *25*, 2474–2480. https://doi.org/10.1002/adma.201205332.
3. Wang, J.; Wu, C.; Dai, Y.; Zhao, Z.; Wang, A.; Zhang, T.; Wang, Z.L. Achieving ultrahigh triboelectric charge density for efficient energy harvesting. Nature Communications 2017, 8, 88. https://doi.org/10.1038/s41467-017-00131-4.
4. Zheng, R.; Chen, Y.; Chi, H.; Qiu, H.; Xue, H.; Bai, H. 3D Printing of a Polydimethylsiloxane/Polytetrafluoroethylene Composite Elastomer and its Application in a Triboelectric Nanogenerator. Acs Applied Materials & Interfaces 2020, 12, 57441-57449. https://doi.org/10.1021/acsami.0c18201.
5. 61. Wang, J.; Wen, Z.; Zi, Y.; Zhou, P.; Lin, J.; Guo, H.; Xu, Y.; Wang, Z.L. All-Plastic-Materials Based Self-Charging Power System Composed of Triboelectric Nanogenerators and Supercapacitors. Advanced Functional Materials 2016, 26, 1070-1076. https://doi.org/10.1002/adfm.201504675.
6. 62. Wang, S.; Zi, Y.; Zhou, Y.S.; Li, S.; Fan, F.; Lin, L.; Wang, Z.L. Molecular surface functionalization to enhance the power output of triboelectric nanogenerators. Journal of Materials Chemistry A 2016, 4, 3728-3734. https://doi.org/10.1039/C5TA10239A.
7. 63. Zi, Y.; Wu, C.; Ding, W.; Wang, Z.L. Maximized Effective Energy Output of Contact-SeparationTriggered Triboelectric Nanogenerators as Limited by Air Breakdown. Advanced Functional Materials 2017, 27, 1700049. https://doi.org/10.1002/adfm.201700049.
8. 64. Soin, N.; Zhao, P.; Prashanthi, K.; Chen, J.; Ding, P.; Zhou, E.; Shab, T.; Ray, S.C.; Tsonos, C.; Thundat, T.; et al. High performance triboelectric nanogenerators based on phase-inversion piezoelectric membranes of poly(vinylidene fluoride)-zinc stannate (PVDF-$ZnSnO_3$) and polyamide-6 (PA6). Nano Energy 2016, 30, 470-480. https://doi.org/10.1016/j.nanoen.2016.10.040.
9. 65. Tao, X.; Jin, H.; Ma, M.; Quan, L.; Chen, J.; Dong, S.; Zhang, H.; Lv, C.; Fu, Y.; Luo, J. Significantly Enhanced Performance of Triboelectric Nanogenerator by Incorporating BaTiO3 Nanoparticles in Poly(vinylidene fluoride) Film. Physica Status Solidi a-Applications and Materials Science 2019, 216, 1900068. https://doi.org/10.1002/pssa.201900068.
10. 66. Baig, M.M.; Saqib, Q.M.; Noman, M.; Sheeraz, M.; Rasheed, A.; Yousuf, M.; Lee, E.; Kim, J.; Ko, Y.; Patil, C.S.; et al. Novel Intercalation Approach in MXene Using Modified Silica Nanospheres to Enhance the Surface Charge Density for Superior Triboelectric Performance. Advanced Functional Materials 2024, 34, 2408271. https://doi.org/10.1002/adfm.202408271.
11. 67. Gunasekhar, R.; Reza, M.S.; Kim, K.J.; Prabu, A.A.; Kim, H. Electrospun PVDF/aromatic HBP of 4th gen based flexible and self-powered TENG for wearable energy harvesting and health monitoring. Scientific Reports 2023, 13, 50231. https://doi.org/10.1038/s41598-023-50231-z.
12. 68. Yang, W.; Chen, H.; Wu, M.; Sun, Z.; Gao, M.; Li, W.; Li, C.; Yu, H.; Zhang, C.; Xu, Y.; et al. A Flexible Triboelectric Nanogenerator Based on Cellulose-Reinforced MXene Composite Film. Advanced Materials Interfaces 2022, 9, 2102124. https://doi.org/10.1002/admi.202102124.
